# Combining acute alcohol administration with accelerated prefrontal theta burst stimulation: A pilot study of feasibility and inhibitory control

**DOI:** 10.1016/j.dadr.2026.100466

**Published:** 2026-07-28

**Authors:** Michael J. Wesley, William J. McGarrigle, Mark T. Fillmore

**Affiliations:** a Department of Behavioral Science, College of Medicine, University of Kentucky, United States; b Department of Psychology, College of Arts and Sciences, University of Kentucky, United States; c Department of Psychiatry, College of Medicine, University of Kentucky, United States

**Keywords:** Alcohol, Neuromodulation, Theta burst stimulation, Response inhibition, Cognitive control

## Abstract

**Introduction:**

Acute alcohol intoxication impairs inhibitory control. Intermittent theta-burst stimulation (iTBS) of the dorsolateral prefrontal cortex may alter executive control, but little is known about whether accelerated iTBS can be combined with controlled alcohol administration.

**Methods:**

This double-blind, sham-controlled pilot study evaluated feasibility and preliminary behavioral effects of accelerated left prefrontal iTBS during acute alcohol intoxication in healthy young adults (N = 10). Participants completed a Cued Go/No-Go task at baseline and near peak breath alcohol concentration after active or sham iTBS delivered in two bouts separated by 30 min.

**Results:**

All participants in the analytic sample completed both stimulation conditions without serious adverse events or stimulation-related discontinuations. Alcohol increased inhibitory failures relative to baseline. Preliminary analyses suggested greater alcohol-induced increases in inhibition failures following active versus sham iTBS.

**Conclusion:**

Accelerated prefrontal iTBS can be implemented during controlled acute alcohol intoxication in a laboratory setting. Although the behavioral findings are not conclusive, they are hypothesis-generating and underscore the need to evaluate state-dependent neuromodulation effects in larger, balanced studies.

## Highlights

• Accelerated iTBS was delivered during acute alcohol intoxication.
• Alcohol increased inhibitory failures on a Cued Go/No-Go task.
• Active iTBS altered alcohol-related inhibitory control.
• The combined alcohol and iTBS protocol was tolerated.
• Larger balanced studies are needed to test state-dependent effects.

## 1 Introduction

Alcohol impairs executive functioning, including inhibitory control, contributing to increased drinking and other adverse consequences (Day et al., 2015, Fillmore, 2003, Guillot et al., 2010, Vonghia et al., 2008, World Health Organization, 2026). Laboratory studies consistently show that alcohol increases inhibition failures on cognitive-control tasks (Fillmore et al., 2005, Fillmore et al., 2008, Fillmore and Weafer, 2012, Marczinski et al., 2007, Miller and Fillmore, 2014, Roberts et al., 2013). These impairments may reflect altered function within prefrontal networks, including the dorsolateral prefrontal cortex (DLPFC) and connected regions supporting executive control, working memory, and goal-directed behavior (Bickel et al., 2014, Rossi et al., 2009, Wesley and Bickel, 2014, Wesley et al., 2014, Wesley et al., 2016, Wesley et al., 2017). The DLPFC interacts with regions involved in conflict monitoring and action selection, including the dorsal anterior cingulate cortex, as part of a distributed cognitive-control network (Badre and Wagner, 2004, Bellgrove et al., 2004, Carter and van Veen, 2007, Hester et al., 2004).

Transcranial magnetic stimulation (TMS) permits causal testing of cortical contributions to behavior (Hallett, 2007, Pascual-Leone et al., 2000). Intermittent theta burst stimulation (iTBS) is often characterized as excitatory, although its effects vary by dose, spacing, and brain state, and higher pulse doses may produce effects opposite to standard protocols (Di Lazzaro and Pilato, 2008a; Di Lazzaro and Pilato, 2008a, Di Lazzaro and Ziemann, 2008b, Huang et al., 2005, McCalley et al., 2021). Under sober conditions, prefrontal stimulation has been associated with improved executive control and working memory (Barr et al., 2009, Guse et al., 2010, Luber and Lisanby, 2014). Accelerated protocols deliver multiple stimulation blocks over a short interval and may produce more durable effects (Caulfield et al., 2022, Cole et al., 2020, Steele et al., 2019, van Rooij, et al., 2023, Zuschlag et al., 2023). For example, iTBS administrations separated by approximately 30 min produced more sustained motor-cortical excitability than a single administration (Yu et al., 2020).

Acute alcohol-related changes in cortical excitability and network dynamics may alter the behavioral effects of stimulation (Kahkonen et al., 2003, Shokri-Kojori et al., 2017, Su et al., 2025, Ziemann et al., 1995). If alcohol-induced disinhibition reflects reduced prefrontal control, iTBS might counteract this effect; alternatively, the alcohol-altered brain state could modify the stimulation response. Despite growing interest in accelerated stimulation and neuromodulation for alcohol-related disorders (Ekhtiari et al., 2019, McCalley et al., 2022, Philip et al., 2020), no published study has examined accelerated iTBS during controlled alcohol intoxication in humans.

This pilot study evaluated the feasibility and preliminary behavioral effects of accelerated left-DLPFC iTBS during controlled alcohol intoxication. Healthy young adults completed a within-subject, double-blind comparison of alcohol plus active versus sham iTBS. We assessed protocol tolerability and alcohol-induced impairment on a validated Cued Go/No-Go task. We hypothesized that alcohol would impair inhibitory control relative to baseline and that this impairment would be attenuated by active versus sham iTBS.

## 2 Methods

The study included three visits to the Neurobehavioral Systems Lab (NSL) at the University of Kentucky (UK). All procedures were approved by the UK Institutional Review Board (Protocol #: 92383), and participants provided written informed consent. Visit 1 included consent, resting motor threshold (RMT) assessment, TMS familiarization, and task training. Visits 2 and 3 were double-blind, sham-controlled alcohol/TMS test sessions separated by at least two days.

**Participants.** Individuals aged 21–29 years were recruited locally. Inclusion required social alcohol use and no neurological or psychiatric disorder; exclusions included seizure or significant head-injury history, TMS contraindications, psychoactive medications, and alcohol use disorder. Twenty individuals consented. Six did not proceed to testing (three prescribed SSRIs, two unable to tolerate iTBS during familiarization, and one unable to provide the Social Security number required for payment). Fourteen completed both experimental visits; three lacked valid Go/No-Go data and one vomited after alcohol during the second visit before TMS administration. The analytic sample included 10 completers with usable data (ages 21–24 years; 5 m, 5 f).

**Familiarization and Training (Visit 1).** Resting motor threshold (RMT) for TMS was determined using the PEST algorithm (Borckardt et al., 2006). Participants received a single bout (900 pulses) of intermittent theta-burst stimulation (iTBS) targeting the left dorsolateral prefrontal cortex (DLPFC), localized using the 5.5 cm rule. Participants were then trained on the Cued Go/No-Go task. Alcohol was not administered during this visit.

**Fig. 1:**
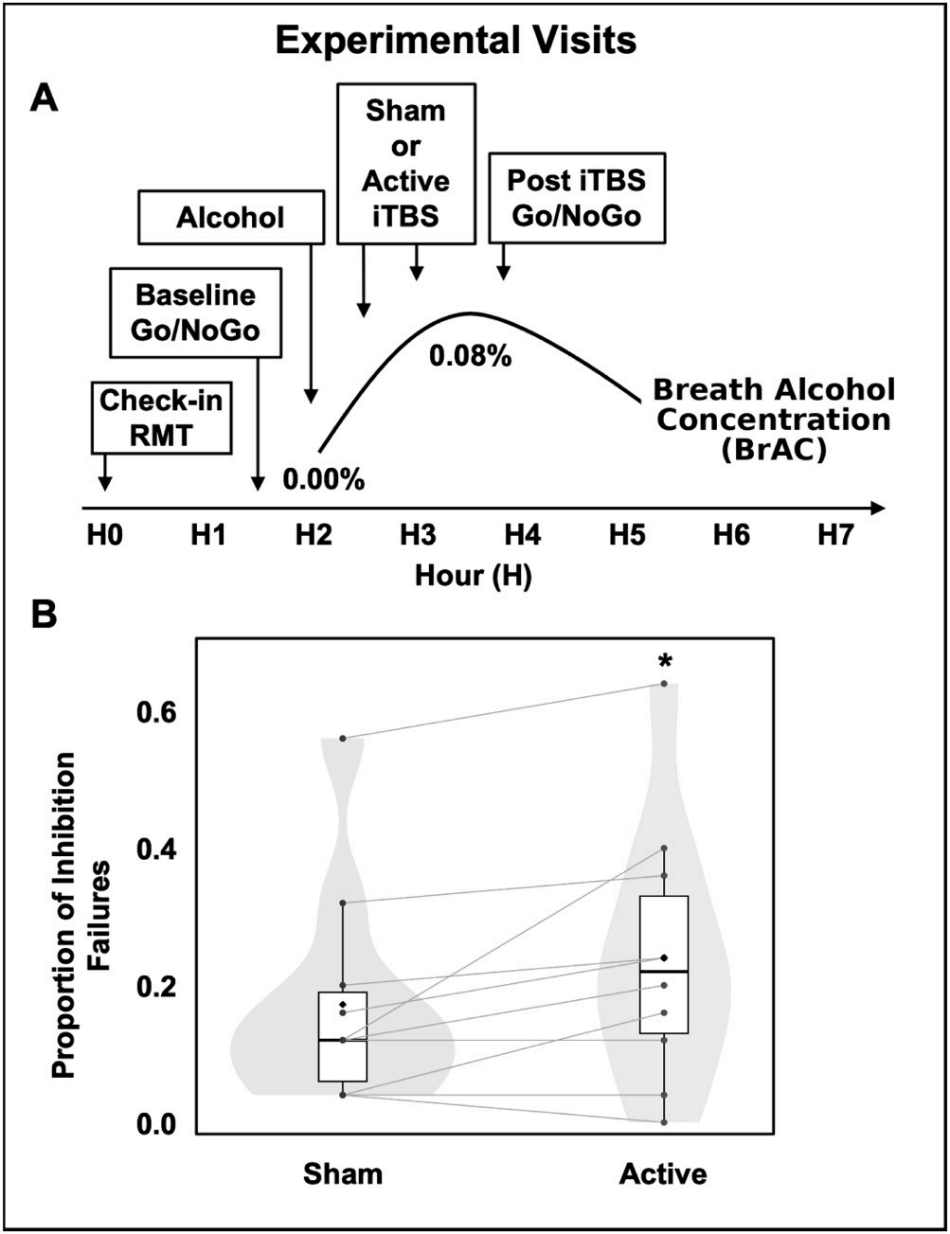
Experimental design and behavioral effects of accelerated iTBS during acute alcohol intoxication. A. Participants completed baseline inhibitory-control testing before consuming alcohol (0.60 g/kg for women; 0.65 g/kg for men). As BrAC ascended toward the expected peak (~0.08%), they received two administrations of active or sham iTBS targeting the left DLPFC, separated by 30 min. Post-stimulation testing occurred near peak intoxication. B. Alcohol-induced change in inhibition failures under sham and active iTBS. Violin plots show distribution density with embedded boxplots; paired lines represent individual participants. ⁎p = .038 for the planned active-versus-sham comparison.

**Alcohol and TMS (Visits 2 and 3).** Participants were informed they could receive active or sham stimulation and real or placebo alcohol. All received active alcohol at both experimental visits paired with active or sham accelerated iTBS; order was assigned using a random-number generator. Fig. 1A summarizes test-day procedures. Participants fasted for four hours and abstained from alcohol and other substances for 24 h, verified by survey, breathalyzer, and urine drug screen. A formal alcohol-withdrawal scale was not administered; all arrived with BrAC = 0.000%, and no participant displayed or reported concerning withdrawal symptoms.

Baseline inhibitory control was assessed at both visits. Participants consumed alcohol (0.60 g/kg women; 0.65 g/kg men) mixed with three parts non-caffeinated soda within 10 min. BrAC was measured regularly using a Dräger Alcotest 5820 breathalyzer. During ascending BrAC, participants received active or sham iTBS. Post-stimulation testing occurred approximately 60 min after drinking, near the expected 0.08% peak (Fillmore and Vogel-Sprott, 1999, Marczinski and Fillmore, 2003). Participants remained at the laboratory until BrAC was ≤ 0.02% and they passed a standardized field sobriety test administered by trained study staff; transportation was provided.

**Inhibitory Control Task.** Inhibitory control was assessed using a validated Cued Go/No-Go task (Fillmore et al., 2008, Fillmore et al., 2005, Fillmore and Weafer, 2012, Marczinski et al., 2007, Miller and Fillmore, 2014, Roberts et al., 2013). Participants were instructed to respond as fast as possible to Go targets and to withhold responses to No-Go targets. Preceding cues established response prepotency. The primary outcome measure was the proportion of inhibition failures (p-inhibition) following Go cues. Reaction time (ms) to Go targets was also recorded.

**TMS Protocol.** Stimulation was delivered using a MagVenture X100 device (MagVenture A/S, Farum, Denmark). Resting motor threshold (RMT) was determined using an MC-B60 figure-of-eight coil. The DLPFC target was marked 5.5 cm anterior to the left motor hotspot along a parasagittal plane. Intermittent theta burst stimulation (iTBS) was administered at 110% of RMT using a Cool-B65 A/P (active/placebo) figure-of-eight coil positioned over the marked target.

Each experimental visit included two administrations of either active or sham iTBS separated by approximately 30 min, constituting an accelerated protocol. Within each administration, stimulation began with a ramping phase of approximately 300 pulses during which intensity increased from 80% to 110% of RMT. This was followed by 600 pulses delivered at 110% RMT, resulting in 900 total pulses per administration (1800 pulses per experimental visit). Administration followed standard patterned parameters, consisting of bursts of three pulses at 50 Hz repeated at 5 Hz (Huang et al., 2005).

Sham stimulation was delivered using the integrated placebo mode of the Cool-B65 A/P coil. Identical coil positioning and acoustic characteristics were maintained while minimizing cortical stimulation. Surface electrodes were positioned approximately 1 cm apart on the scalp beneath the stimulation cap to deliver mild electrical stimulation. Participants and experimenters conducting behavioral testing were blind to stimulation condition.

**Subjective Alcohol Effects.** Immediately before post-alcohol testing, 100-mm visual analog scales assessed liking, stimulation, sedation, desire for more alcohol, and intoxication (0 = not at all; 100 = very much).

**Statistical Analysis.** A 2 (Alcohol: baseline vs. intoxication) x 2 (Stimulation: active vs. sham) repeated-measures ANOVA examined inhibition failures, followed by planned paired comparisons. Effect sizes are partial eta squared (ηp²) and Cohen’s d; p < .05 was significant. Peak BrAC and subjective effects were compared using paired *t*-tests. Feasibility was defined as completion of both stimulation administrations, behavioral testing, discharge procedures, and both visits. Tolerability was assessed by open-ended symptom checks and willingness to continue after each administration and at session end.

## 3 Results

**Feasibility and Tolerability.** Fourteen participants completed both experimental visits. Of these, three did not pass Go/No-Go data validation because their error rates approached chance-level responding, and one vomited after alcohol administration during the second visit; these four participants were not included in the analytic sample. The remaining 10 participants completed both stimulation conditions, behavioral testing, and discharge procedures. Seven received alcohol plus active iTBS during their first experimental session, and all seven returned for the second session. All participants indicated that they were willing to continue after each stimulation administration, and none requested discontinuation. Commonly reported sensations included tingling, pinching, and knocking. No serious adverse events or stimulation-related discontinuations occurred among the 10 participants included in the analytic sample.

**Breath Alcohol Concentration and Subjective Effects.** Peak BrAC was within the targeted range (~0.08%) and did not differ significantly between active (M = 0.067%, SD = 0.006) and sham (M = 0.063%, SD = 0.010) stimulation sessions, t(9) = −1.09, p = .299, d = 0.35. Ratings of liking, stimulation, sedation, desire for more alcohol, and intoxication did not differ between stimulation conditions (*p*s ≥ .250).

**Inhibitory Control.** Fig. 1B shows the mean change from baseline in inhibition failures under alcohol following sham and active iTBS. Alcohol significantly increased inhibitory failures relative to baseline, indicating impaired inhibitory control under intoxication, F(1, 9) = 29.90, p < .001, ηp² = .77. Fig. 1B also shows greater pre- to post-alcohol increases in inhibition failures following in active versus sham iTBS. Despite a large effect size, this Alcohol ×  Stimulation interaction did not reach conventional significance, F(1, 9) = 4.29, p = .068, ηp².

= .32. However, a planned comparison of the alcohol-induced change between the TMS conditions confirmed a significantly greater increase in inhibition failures following active versus sham stimulation, t(9) = 2.43, p = .

.038, d = 0.77 (95% CI [0.04, 1.46]). Thus, contrary to our initial hypothesis, iTBS exacerbated alcohol-induced disinhibition. Reaction time to Go targets did not differ significantly as a function of Alcohol or Stimulation (*p*s ≥ .250).

## 4 Discussion

DLPFC-targeted iTBS provides a causal approach for testing whether modulation of prefrontal control networks alters alcohol-induced disinhibition. This pilot study shows that controlled alcohol administration can be combined with accelerated prefrontal iTBS and that the protocol was tolerated among participants in the analytic sample, with no serious adverse events or stimulation-related discontinuations. Consistent with our previous studies, alcohol significantly impaired inhibitory control, as reflected by increased inhibition failures (Fillmore et al., 2005, Fillmore et al., 2008, Fillmore and Weafer, 2012, Marczinski et al., 2007, Miller and Fillmore, 2014, Roberts et al., 2013).

Preliminary behavioral results suggested that active iTBS increased, rather than attenuated, alcohol-induced inhibitory failures. This was unexpected given the common characterization of iTBS as excitatory and the role of the DLPFC in cognitive control. One possible explanation is that the stimulation protocol produced nonlinear or dose-dependent effects. Prior work suggests that iTBS pulse number can alter the direction of cortical excitability effects; specifically, 1200 pulses produced an inhibitory rather than facilitatory response in the motor cortex (McCalley et al., 2021). The current protocol delivered 900 pulses twice per session, including a ramp period, and therefore may not be equivalent to a standard single 600-pulse iTBS protocol. Alcohol-related changes in cortical excitability and network state may have further shaped the stimulation response (Kahkonen et al., 2003, Ziemann et al., 1995).

These findings require caution because the small sample may yield inflated or unstable effect sizes. Seven participants received active stimulation first, so order effects cannot be excluded, and peak BrAC was modestly higher during active sessions. The study lacked a separate sober iTBS condition and standardized withdrawal assessment; blinding integrity and physiological target engagement were also not assessed.

In summary, this pilot study demonstrates that accelerated iTBS during acute alcohol intoxication can be implemented under controlled laboratory conditions and yields preliminary evidence of stimulation-related modulation of alcohol-induced disinhibition. Larger studies with balanced order, formal blinding assessment, physiological measures of stimulation effects, and sober stimulation control conditions are needed to determine whether the observed behavioral pattern reflects a reliable alcohol-by-neuromodulation interaction.

## CRediT authorship contribution statement

**Michael J. Wesley:** Writing – review & editing, Writing – original draft, Visualization, Supervision, Resources, Project administration, Methodology, Funding acquisition, Conceptualization. **William J. McGarrigle:** Writing – review & editing, Methodology, Formal analysis, Conceptualization. **Mark T. Fillmore:** Writing – review & editing, Supervision, Methodology, Funding acquisition, Conceptualization.

## Primary Funding

K01DA043652, T32AA027488 and UL1TR001998.

## Declaration of Competing Interest

The authors declare that they have no known competing financial interests or personal relationships that could have appeared to influence the work reported in this paper.
